# Health and working conditions of pregnant women working inside and outside the home in Mexico City

**DOI:** 10.1186/1471-2458-7-25

**Published:** 2007-02-27

**Authors:** Laura del Pilar Torres-Arreola, Patricia Constantino-Casas, Juan Pablo Villa-Barragán, Svetlana Vladislavovna Doubova

**Affiliations:** 1 Health Services and Epidemiologic Research Unit. National Medical Center Century XXI, Mexican Institute of Social Security, Mexico; 2 Research Unit in Health Economics. Coordination of Health Research. National Medical Center Century XXI. Mexican Institute of Social Security, Mexico; 3 Pediatric National Institute, Secretary of Health, Mexico

## Abstract

**Background:**

To explore differences related to health and working conditions by comparing socio-demographic parameters, reproductive and prenatal care characteristics and working conditions among pregnant women who are employed outside the home (extra-domestic) while still performing a domestic workload versus those who perform exclusively domestic work in the home (intra-domestic).

**Methods:**

A cross-sectional study was carried out at Family Medicine Unit N 31 of the Mexican Institute of Social Security (IMSS) in Mexico City between April and July 2003. Interviews were conducted with 537 pregnant women engaged in either extra-domestic work plus intra-domestic tasks, or those performing strictly intra-domestic work. Information was obtained regarding their demographic status, prenatal care, reproductive, work characteristics, and health during pregnancy.

**Results:**

One hundred ninety-six (36.5%) of the interviewed women had paid jobs outside the home in addition to domestic tasks, while three hundred forty-one (63.5 %) engaged in exclusively intra-domestic occupations. Of the women with paid jobs, 78.6% worked as clerks. Among domestic tasks, we found that the greatest workload was associated with washing of clothes, and our micro-ergonomic analysis revealed that women who worked strictly inside the home had a higher domestic workload versus employed women (69.2 vs. 44.9%). When we analyzed the effect of work on health during pregnancy, we observed that women who worked strictly inside the home were at a higher risk for musculoskeletal and genitourinary symptoms than those employed outside the home.

**Conclusion:**

These findings suggest that the effect of intra-domestic work should not be ignored when considering women's health during pregnancy, and that greater attention should be paid to women's working conditions during intra and extra-domestic work.

## Background

Over the past few decades, the participation of women in the workplace has increased in Mexico and worldwide [1,2], meaning that more women of reproductive age are now employed outside the home (extra-domestic), while still being expected to fulfill traditional (intra-domestic) familial roles [3].

To date, most studies on the working conditions and health of women (inclusively pregnant women) have focused more on extra-domestic risks than those associated with intra-domestic work. Certain working conditions (e.g. poor illumination, ventilation, temperature, among others that generate labor fatigue and stress at work) have been shown to trigger adverse results in both mother and newborn [4-12], leading to the suggestion that pregnant women should reduce their working hours or switch to less strenuous work. However, other studies have shown that instead of diminishing the extra-domestic workload, 'some women increase their' workload during pregnancy [13].

Unlike extra-domestic work, which has an inherent monetary value, intra-domestic work is often vastly undervalued [14]. As such, relatively few studies have addressed health risks to pregnant women performing intra-domestic work. The few studies that have addressed such matters suggest that the physical risks due to household tasks include heavy lifting and the use of irritating substances that could produce musculoskeletal and reproductive damage, as well as poisonings and contact dermatitis [15]. Clearly, additional studies are warranted in terms of the health risks to women exposed to both extra- and intra-domestic workloads.

Accordingly, we herein compared the socio-demographic parameters, reproductive and prenatal care characteristics and working conditions among pregnant women who perform extra-domestic work plus traditional domestic duties, versus those engaged in exclusively intra-domestic work.

## Methods

We carried out a cross-sectional study in Family Medicine Unit N 31 of the Mexican Institute of Social Security (IMSS; Mexico City) between April and July 2003. Five-hundred and thirty-seven pregnant women were interviewed, using a questionnaire designed to obtain information on the interviewee's demographic status, reproductive, prenatal care and work characteristics, and health during pregnancy. This questionnaire was designed in collaboration with experts on ergonomics, occupational health, sanitation, gynecology, and reproductive health.

Within the questionnaire, extra-domestic work was assessed using variables related to the type and branch of activity, as well as workplace characteristics. Domestic work was characterized in terms of the performed tasks and the conditions and limitations that the women faced during daily domestic activities.

A micro-ergonomic index (low, average and high workload) was built with three levels of domestic workload by integrating each activity (laundry, ironing cooking, sweeping/dusting, cleaning bathrooms, washing windows, washing dishes, shopping, etc.) combining the frequency of the activities per day, the time invested in each of them, and the use of electronic domestic equipment (washing machine, vacuum etc).

For evaluation of health related conditions, the interview included indicators for symptoms associated with ocular, auditory, genitourinary and musculoskeletal distress. Each indicator was assessed as 'damage' or 'no damage.'

In a pilot study, 20 pregnant women were given the questionnaire, and its ease of use and time for application were evaluated.

During the study the interviews were given by trained nurses, and informed consent was obtained. The data were analyzed using descriptive statistics. Variables were compared between groups using the chi-squared or Fisher's exact tests for discreet variables, and the t-test for continuous variables. Logistic regression analysis was used to evaluate the effect of working conditions on the health of the pregnant women. All statistical analyses were performed using the Stata 8.0 software package (Stata Corp, College Station, TX). The study has a 90% power, assuming an alpha of 0.05 (one-side test) for a 15% group-specific difference in health conditions.

This research was carried out in compliance with the Helsinki Declaration and with the approval of an appropriate ethics committee (IMSS national wide), registration number: 2001-785-013.

## Results

Of the 537 pregnant women interviewed, 36.5% were employed in extra-domestic work plus a domestic workload (A), while 63.5% were exclusively intra-domestic in their work (B). The median age of interviewees was 24 years (range, 14–40 years). All enrolled women could read and write, but there were intra-group differences in terms of formal schooling. The women of group B had attended school for a median of 9 years (range 0–17 years), while those of group A had attended school for a median of 12 years (range 3–17 years). We also observed differences in the socioeconomic level; the women in group A had a higher socioeconomic level (30.6% vs.25,2%, *p *< 0.05), including better housing conditions (e.g. better construction and potable water within the house) and a greater percentage of automobiles (26.5% vs.13.8%) compared with the women in group B (*p *< 0.05) (Table 1). In terms of family structure, more women in group B lived with their spouses (81.0% vs.54.6%, *p *< 0.05), who were most often the head of household. The average number of family members among all interviewees was three. With regard to some of the women's habits we found a greater number of women in group A smoked prior to and/or during their pregnancies, compared with the women in group B (23.0% vs. 18.7% and 11.1% vs. 4.7%, respectively; *p *< 0.05). In contrast, while alcohol consumption was low during pregnancy in both groups, this parameter was higher in group B (2.05 vs. 0.51%, respectively). In terms of the reproductive characteristics and the prenatal care, we found a greater proportion of null parity among the women in group A (47.4% vs. 3.3%, *p *< 0.05). A greater proportion of women from group A attended prenatal care appointments during the first trimester of their pregnancies (71.5% vs. 62.7, *p *< 0.05), compared to group B, while a relatively large proportion of women from group B (13.5% vs.8.0%, *p *< 0.05) failed to initiate prenatal care until the third trimester compared to group A (Table 1).

**Table 1:** Characteristics of the study population

**Characteristics**	**Extra-domestic work and additional domestic workload (A) *n *= 196**		**Exclusively intra-domestic work (B) *n *= 341**	
	**Frequency**	**%**	**Frequency**	**%**

**I. Socio-demographic characteristics**				
Age (years)*				
14–19	17	8.7	52	15.3
20–34	169	86.2	279	81.8
≥ 35	10	5.1	10	2.9
Marital status*				
Married	107	54.6	276	81.0
Free union	60	30.6	57	16.7
Single, divorced	29	14.8	8	2.3
	*n *= 196		*n *= 340	
Education (years)*				
≤ 3	2	15.3	6	1.8
4–6	19	41.6	46	13.5
7–9	59	34.9	142	41.8
10–12	77	7.9	119	35.0
> 12	39	19.9	27	7.9
Socio-economic level*				
High	60	30.6	86	25.2
Medium	91	46.4	135	39.6
Low	45	22.9	120	35.2
**Goods**				
House	99	50.5	162	47.5
Car*	52	26.5	47	13.8
Washing machine	128	65.3	213	62.5
Stove	189	96.4	330	96.8
Refrigerator*	165	84.2	260	76.3
Computer*	39	19.9	37	10.8
Television	191	97.4	329	96.5
Reproductive and prenatal care characteristics Parity*				
1	93	47.4	124	36.3
2–3	92	46.9	189	55.4
> 3	11	5.6	28	8.2
Failure to attend prenatal care*	3	1.5	30	8.8
**First prenatal care visit**	*n *= 193		*n *= 311	
First trimester*	138	71.5	195	62.7
Second trimester	47	24.3	74	23.8
Third trimester*	8	4.1	42	13.5

*P *< 0.05

We then focused on the working conditions of the pregnant women. Of the women in group A, the vast majority worked as clerks (78.6%); of them, 44% worked in the services, and 12.3% worked in administration. We found that the majority of women in group A worked outside the home to contribute to the family income (73.5%), with 12.7% of the group A respondents reporting that they were the sole source of income for the family. An additional 13.8% of the women in group A reported working for the purpose of professional development. Of the women in group A, 82% reported unfavorable working conditions, including poor illumination (80.1%), ventilation (52%) and temperature (50.5%). In addition, 74% of the women in group A reported having stress at work. When we assessed the work situation further, we found that 25% of the women in group A worried that their pregnancies might lead to job termination; one of these women had already been dismissed once her boss learned of her pregnancy, while three others reported that they had not informed their employers of the pregnancy. Of the women in group A, 82% reported being unaware of their working rights during pregnancy. Among those who claimed to know their rights, most reported only knowledge about maternity leave, and access to medical care (Table 2).

**Table 2:** Working characteristics of women dedicated to extra-domestic work

**Characteristics**	**Extra-domestic work *n *= 196**	
	**Frequency**	**%**

**Type of activity**		
Clerk	154	78.6
Unskilled worker	37	18.9
Independent work	5	2.5
**Branch of activity**		
Administrative	52	33.8
Manufacturing	19	12.3
Services	69	44.8
Domestic services	14	9.1
**Workplace characteristics**		
Inadequate illumination	157	80.1
Inappropriate temperatures	99	50.5
Inadequate ventilation	102	52.0
Noise	81	41.3
**Stress at work**	145	74.0
**Stress by type of work**		
Independent professional	4	2.7
Clerk	115	79.3
Unskilled worker	26	18.0
**Stress by branch of activity**		
Administrative	36	31.3
Manufacturing	12	10.4
Services	61	53.0
Domestic services	6	5.2
**Reason for extra-domestic work**		
Contribution to family income	144	73.5
Professional development	27	13.8
Sole source of income for the family	25	12.7
**Changes at work due to pregnancy**		
Job change due to pregnancy	18	9.2
Job insecurity or dismissal due to pregnancy	50	25.5
Absence of knowledge about women's maternity rights	161	82.0

When we assessed the working conditions of the women in both groups when engaged in traditional domestic activities we found that the greatest workload of women in group B was associated with washing of clothes, washing dishes, cleaning of baths and cooking. Interestingly, our micro-ergonomic domestic indicator analysis revealed that the women of group B had a higher domestic workload than those in group A (69.2% vs. 44.9%, respectively: *p *< 0.05) (Table 3).

**Table 3:** Characteristics of household activities at home

**Characteristics**	**Extra-domestic work (A) *n *= 196**		**Exclusively intra-domestic work (B) *n *= 341**	
	**Frequency**	**%**	**Frequency**	**%**

**Type of activity**				
Washing clothes*^+^	92	46.9	211	61.8
Ironing	48	24.5	96	28.1
Cooking*	25	12.8	115	33.7
Sweeping/dusting*	58	29.6	165	48.3
Cleaning bathrooms *	44	22.4	130	38.1
Making beds	49	25.0	102	29.9
Cleaning stove and/or refrigerator*	58	29.6	125	36.6
Washing windows*	21	10.7	58	17.0
Washing dishes*	65	33.1	167	48.9
Shopping *	49	25.0	166	48.7
Micro-ergonomic domestic indicator *	88	44.9	236	69.2

*p < 0.05

^+^High domestic workload was calculated in terms of frequency of the activity over time.

Finally, when we compared the health of women in groups A and B, we found that more women in group B reported genitourinary (such as dysuria, frequency and urgency, straining to urinate) and musculoskeletal symptoms (such as lower limb and back pain, stiffness, general movement restriction) versus those in group A (61.8% vs. 54.0% and 81.8% vs. 75.5%, respectively: *p *< 0.05), while reports of cardiovascular symptoms were similar in both groups (Table 4). Interestingly, younger women (< 20 years) in group B reported musculoskeletal symptoms more often than women of the same age range in group A (OR: 4.3: IC95% 1.6–11.4). Genitourinary symptoms were observed more frequently in women in group B with three or more children (OR: 3.05: IC95% 1.3–7.3) (Table 5).

**Table 4:** Health damage indicators during pregnancy

**Indicator**	**Extra-domestic work (A)** ***n *= 196**		**Exclusively intra-domestic work (B) ** ***n *= 341**		***p*-value**
	**Frequency**	**%**	**Frequency**	**%**	

**Eye**	104	53.0	192	56.3	0.5
**Hearing**	31	15.8	50	14.6	0.7
**Cardiovascular**	122	62.2	208	61.0	0.7
**Genitourinary**	106	54.0	211	61.8	0.07
**Musculoskeletal**	148	75.5	279	81.8	0.08

*x^2^

**Table 5:** Health damage indicators related with working conditions

**Variable**	**Indicator**					
	**Musculoskeletal**		**Cardiovascular**		**Genitourinary**	
	OR**	**IC 95%**	OR**	**IC 95%**	OR**	**IC 95%**
**Exclusively intra-domestic work**	1.45	0.7–2.2	1.6	0.8–2.9	1.87*	1.01–3.5
**Micro-ergonomic domestic indicator**						
High workload	1.05	0.6–1.68	0.7	0.41–0.93	0.72	0.5–1.07
**Parity**						
2–3	1.21	0.7–1.98	1.4	0.92–2.14	1.36	0.9–2.05
> 3	2.5	0.84–7.4	1.4	0.62–3.1	3.05*	1.3–7.3
**Stress**	1.17	0.5–2.5	2.03*	1.05–3.9	1.6	0.8–3.06
**Age**						
< 20 years	4.3*	1.6–11.4	1.9	0.91–3.9	1.7	0.87–3.5
20–24 years	1.7*	1.0–2.9	0.94	0.59–1.5	1.6	0.99–2.5

* p < 0.05

** Adjusted by schooling, marital status, history of smoking, and alcohol consumption during pregnancy.

## Discussion

The present study examined differences in health and other characteristics between pregnant women with extra-domestic employment plus traditional domestic duties versus those engaged in exclusively domestic work. Of the enrolled women receiving prenatal care at the IMSS in Mexico City, those engaged in exclusively domestic work tended to be younger, perhaps due to cultural patterns that foster early marriage and procreation, as previously noted in studies from Latin America and the Caribbean [16].

Consistent with previous reports, the women working extra-domestic jobs tended to have a higher level of education [17], and most often reported working in order to contribute to the family income [2,14]. In this way, employment was considered complementary to that of the husband; although in some cases the woman was the sole source of income. Consistent with the findings of other studies [14,18], most of the working women enrolled in the present study were employed in the services, followed by administrative and domestic work. Overall, the working conditions of these women were relatively poor, including inadequate ventilation, illumination and noise, which could easily affect their mental states, generate stress and provoke health problems [10-12]. In addition, approximately half of these women suffered from feelings of job insecurity related to their pregnancies; one woman had already been dismissed and three others were attempting to hide their pregnancies from their employers. This situation shows that although pregnancy is a physiological condition, it may also represent a psychological vulnerability in the workplace [16]. Thus, our present findings underscore the often poor situation of the working woman in Mexico, who is often expected to take a relatively insecure position, with low wages and few benefits to offset the stress of her dual role as both housekeeper and worker. However, it does not seem as though avoiding extra-domestic employment will necessarily improve a pregnant woman's health. For example, our findings were consistent with those of other studies showing that women with extra-domestic jobs tended to have a better socioeconomic level and decreased responsibility for household tasks [19]. In addition, we found that women who worked strictly within their home tended to report more musculoskeletal and genitourinary health problems. However, it should be noted that our study was somewhat limited because these health problems were assessed by self-reporting of the presence or absence of symptoms, giving us only an approximation of a given response's actual health.

Literature has showed that there is a relationship between workload and some pregnancy outcomes, such as abortion, preterm birth and low birth weight; however, in this study we could not evaluate its effect. Longitudinal studies are necessaries to evaluate the relationship of higher workload and reproductive outcomes.

In general, our findings agree with other studies reporting that women who work outside the house have better health versus those who work exclusively in the home [20-22]. One possible explanation for this finding is that working women are often more educated, and may have a better sense of how to lead a healthy lifestyle. This hypothesis is consistent with our observation that more women who worked strictly within the home waited until the third trimester of their pregnancy before seeking prenatal care.

## Conclusion

In sum, we herein showed that Mexican women who worked strictly inside the home had a higher domestic workload versus employed women and a higher risk for musculoskeletal and genitourinary symptoms than those employed outside the home. These findings suggest that the effect of intra-domestic work should not be ignored when considering women's heath during pregnancy, and that greater attention should be paid to women's working conditions during intra and extra-domestic work. The present study provides a useful starting point for identifying potential health risks for pregnant women, and will hopefully encourage new planning efforts with an aim towards diminishing these risks.

## Competing interests

The author(s) declare that they have no competing interests.

## Authors' contributions

LPTA, JPVB contributed in the conception and design of the study and statistical analysis. SD, NPCC reviewed for important intellectual content. All authors participated in the interpretation of data and read and approved the final version to be published.

## Pre-publication history

The pre-publication history for this paper can be accessed here:


